# Laparoscopic Management of an Inflammatory Pseudotumor Mimicking a Locally Advanced Renal Carcinoma: A Diagnostic Pitfall

**DOI:** 10.1155/2022/4485930

**Published:** 2022-02-23

**Authors:** Imad Boualaoui, Idriss Ziani, Salma Marrakchi, Mohammed Raiss, Fouad Zouidia, Ahmed Ibrahimi, Hachem El Sayegh, Yassine Nouini

**Affiliations:** ^1^Department of Urology A, Mohammed V University in Rabat, Ibn Sina University Hospital, Rabat, Morocco; ^2^Department of Radiology, Mohammed V University in Rabat, Ibn Sina University Hospital, Rabat, Morocco; ^3^Surgical Department “C”, Mohammed V University in Rabat, Ibn Sina University Hospital, Rabat, Morocco; ^4^Department of Anatomopathology and Cytology, Mohammed V University in Rabat, Ibn Sina University Hospital, Rabat, Morocco

## Abstract

Inflammatory pseudotumors of the kidney are an infrequent entity. More frequently described in the lung, the genitourinary tract location is rare. Commonly described in the bladder, the kidney damage remains exceptional. Herein, we report the case of 60 years old man with a history of flank pain, initially diagnosed with a locally advanced left renal carcinoma invading the left colon. Then, after performing a laparoscopic radical nephrectomy, the histopathological diagnosis of inflammatory pseudotumor of the left kidney has been made.

## 1. Introduction

Inflammatory pseudotumors of the kidney are a rare entity. More frequently described in the lung, the genitourinary tract location is rare [1]. Commonly described in the bladder, the kidney damage remains exceptional. Few cases have been reported in the literature. Herein, we report the case of 60-year-old man with a history of flank pain, initially diagnosed with a locally advanced left renal carcinoma invading the left colon. Then, after performing a laparoscopic radical nephrectomy, the histopathological diagnosis of inflammatory pseudotumor of the left kidney has been made. This case discussion is aimed at bringing light to this rarely encountered entity. Inflammatory neoplasms of the kidney needed to be kept in mind in cases of atypical radiological presentation of a renal tumor. Appropriate management of renal pseudotumors allows avoiding an unnecessary radical nephrectomy.

## 2. Case Presentation

**Figure 1:**
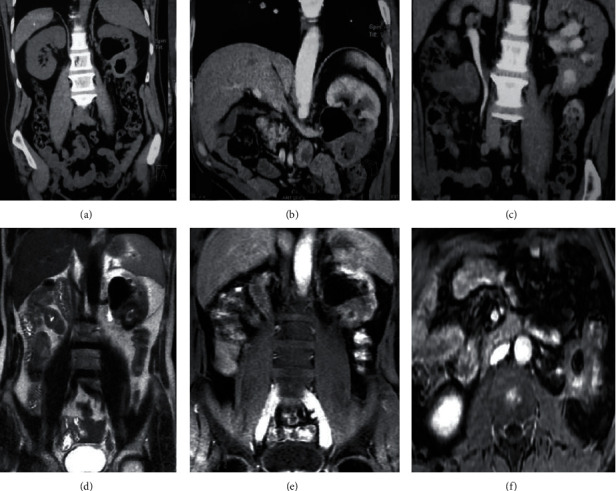
(a–c) Computed tomography; (a) pelvic hydroaeric level in the left kidney; (b) poorly enhanced lower pole in the left kidney; (c) contrast medium extending to the left colon. (d–f) Magnetic resonance imaging; (d) 41 × 38 × 41 mm T2 hypointense lower pole left renal mass associated with a pelvic hydroaeric level; (e) contrast medium extending to the left colon; (f) left renal mass invading the proximal ureter, the left colic angle, and the psoas muscle.

A 60-year-old man has been presented with a year history of apyretic left flank pain. The clinical exam was normal, and the urine was clear. The first computerized tomography has shown a pelvic and caliceal dilatation associated with an aerial content and a hydroaeric level in the left renal pelvis. Moreover, the excretory phase of the uro-CT shows a contrast medium not only in the renal pelvis but also in the left colic angle extending to the transverse colon. These findings confirmed the diagnosis of a left renocolic fistula (Figures 1(a)–1(c)).

To certify the confirmed etiology of the fistula and to understand its genesis, we performed a magnetic resonance urography. It has revealed a 41 × 38 × 41 mm lower pole left renal mass, rounded with irregular contours, and low signal intensity in T2-weighted spin-echo images. Outwards, the renal mass invaded the left colic angle noting the gadolinium flow to the colic lumen certifying the renocolic fistula. Inner, it infiltrates the proximal ureter what is causing 37 mm hydronephrosis. Backward, the mass is closely adhering to the psoas muscle (Figures 1(d)–1(f)).

A 6 mm unique regional lymph node has also been demonstrated. The thoracic computed tomography did not reveal any metastasis. The tumor is then classified as a T4N1M0. We decided to perform a laparoscopic radical nephrectomy with a segmental colic resection.

**Figure 2:**
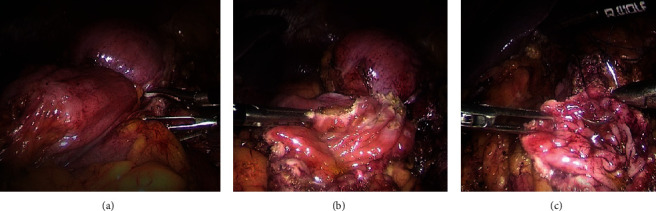
Laparoscopic images; (a) left renal tumor invading the left colon angle; (b) left lateral and segmental colonic resection began with opening the colonic mucosa in a healthy area; (c) closure of the colonic mucosa with a 4-0 vicryl thread.

After bowel preparation, we performed a left lateral and segmental colonic resection. We firstly proceed on lowering the left colon by performing an extensive dissection of the left colon and transverse to the tail of the pancreas medially and the spleen and stomach above. Then, we completed the resection by a total left nephrectomy extending to the perirenal fat and preserving the left adrenal (Figure 2).

**Figure 3:**
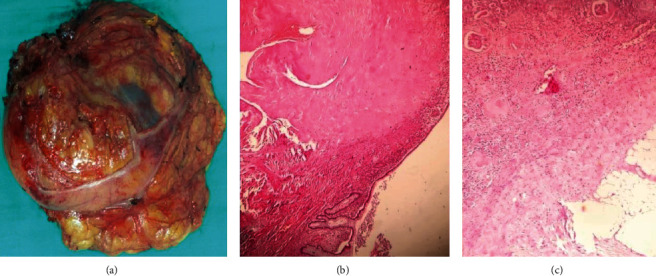
(a) Macroscopic image of the specimen; (b) inflammatory lesion infiltrating the intestinal mucosa (Hemalin-Eosine ×40); (c) renal tumor harboring an inflammatory lymphoplasmacytic infiltrate on a fibrous background (Hemalin-Eosine ×100).

The histopathologic study of the specimen using the Hemalin-Eosine coloration showed an inflammatory pseudotumor of the kidney. The inflammatory infiltrate is composed of lymphocytes, plasma cells, and fibrous backgrounds. The process invades the colic mucosa (Figure 3).

The intestinal transit resumption was effective on the second day after surgery, and the postoperative renal function was normal. The patient did not receive any specific treatment considering the histopathological diagnosis. An enhanced computed tomography has been performed after a month and shown an empty renal lodge without recurrence. We planned to perform an enhanced computed tomography once a year for five years.

## 3. Discussion

Renal inflammatory pseudotumor (RIPT) is an exceptional benign condition. It is also known as inflammatory myofibroblastic tumors, plasma cell granuloma, or myofibroblastoma. Inflammatory pseudotumors appear in the WHO classification as a distinct borderline lesion [2]. Different organs may be affected, especially the lung and orbit, which are the more common locations [1]. Few cases have been reported in the literature. The first urinary tract description was in the renal pelvis by Davides in 1972. However, the bladder is the most commonly described location in the genitourinary tract [3, 4].

The disease occurs at all ages, predominately in children and young adults; both sexes are affected with a slight male predominance [5]. The pathophysiology of these tumors is not yet understood and remains controversial. It is still difficult to determine whether this is reactive hyperplasia or a natural tumor process. The discovery of chromosomal abnormalities suggests that there is also an oncogenetic theory [6].

The etiology of these tumors also remains to be discovered. Different theories have been suggested, including Epstein-Barr and hepatitis B virus infections, and traumatic, vascular, and autoimmune causes, among others. In addition, Schweckendiek et al. also raised the possibility of arrangements of the ALK gene (Anaplastic Lymphoma Kinase), which plays an essential role in the genesis of these tumors [7].

Our patient presented with left lumbar pain without irradiation. The clinical presentation is atypical. Symptoms could be localized pain in the lumbar region like our patient and also renal colic related to hydronephrosis due to ureteral or pelvic compression. However, hematuria is the most frequently described clinical sign in the literature. It can be micro or macroscopic, concomitant, or not with the painful symptoms. It should also be noted that inflammatory pseudotumors can be completely asymptomatic and therefore discovered fortuitously.

Imaging of inflammatory pseudotumors is also nonspecific. This fact explains why in almost all the writings on this subject, the diagnosis is only made on a piece of enlarged nephrectomy. In addition, the radiological signs are misleading, and the diagnosis of a malignant renal tumor is most often difficult to rule out due to the heterogeneous characteristics of the mass. Some characteristics have nevertheless been mentioned in the literature. On ultrasound, the lesion appears rather hypoechoic and heterogeneous, mimicking an atypical cyst. On CT, the lesion is rather hypodense and appears as T2 hypointense on MRI [8].

There is currently no consensus on the management and the follow-up of inflammatory pseudotumors. Due to the rarity of renal involvement, there are no prospective studies on this subject to date. The published articles relate only to case reports.

An atypical presentation for a renal tumor should attract the attention of clinicians, especially in cases of a unique kidney, to avoid unnecessary radical nephrectomies.

## 4. Conclusion

Inflammatory pseudotumors or inflammatory myofibroblastic kidney tumors remain rare, often confused with malignant kidney tumors. Larger-scale studies are needed to have more answers concerning the etiology and pathogenesis of this entity and establish diagnostic criteria in order to limit the number of unnecessary nephrectomies. The rarity of renal involvement, the heterogeneity of the clinical presentation, and the nonspecificity of the radiological signs make it a significant diagnostic and therapeutic challenge and a diagnostic pitfall to be avoided. The laparoscopic approach that we also lift allows a less invasive approach for these lesions whose neoplastic origin has not yet been demonstrated.
